# Bacterial Diversity and Community Assembly in Deep-Sea Sediments: Observations from the Philippine Sea

**DOI:** 10.3390/microorganisms14102129

**Published:** 2026-09-22

**Authors:** Cheng Yang, Dong Sun, Yongji Huang, Mingyan Lai, Li Zhang, Hao Yin, Yanqun Li, Lina Lyu, Si Zhang

**Affiliations:** 1 State Key Laboratory of Tropical Oceanography, South China Sea Institute of Oceanology, Chinese Academy of Sciences, Guangzhou 510301, China; yangcheng22@mails.ucas.ac.cn (C.Y.);; 2 University of Chinese Academy of Sciences, Beijing 100049, China; 3 Key Laboratory of Marine Ecosystem Dynamics, Second Institute of Oceanography, Ministry of Natural Resources, Hangzhou 310012, China; 4 Southern Marine Science and Engineering Guangdong Laboratory (Guangzhou), Guangzhou 511458, China; 5 Guangxi Key Laboratory of Beibu Gulf Marine Resources, Environment and Sustainable Development, Fourth Institute of Oceanography, Ministry of Natural Resources, Beihai 536000, China; 6 Key Laboratory of Tropical Marine Ecosystem and Bioresource, Fourth Institute of Oceanography, Ministry of Natural Resources, Beihai 536000, China

**Keywords:** Philippine Sea, deep-sea sediment, microbial diversity, bacterial community, community assembly

## Abstract

Microorganisms are important components of marine ecosystems and are widely distributed across global marine sediments. Although studies on microbial communities in deep-sea sediments have been increasing worldwide, research in the Philippine Sea is still at an early stage. In this study, we investigated the diversity and composition of bacterial communities in deep-sea sediments of the Philippine Sea and their influencing factors. The results revealed that Proteobacteria dominated the sediment bacterial communities, with clear vertical variations in community composition. Notably, bacterial communities in the shallow and deep sediments of the Parece Vela Rift showed marked differences in community composition, including variation in the abundance of taxa previously reported from hydrate- and seep-associated environments (e.g., Aerophobota), suggesting the possible influence of distinct subsurface environmental conditions. Community assembly analyses indicated that stochastic processes predominated across all four sediment cores, with deterministic processes accounting for only 12.3–21.0%, while community composition varied along measured environmental gradients, and sediment Fe showed an apparent association with phylogenetic turnover. These findings provide baseline data for understanding the mechanisms underlying the formation and maintenance of microbial diversity in deep-sea sediments and offer a reference for exploring the ecological functions of deep-sea ecosystems.

## 1. Introduction

Deep-sea sediments cover more than two-thirds of the Earth’s surface and provide important habitats for microbial communities [1,2,3]. These microorganisms play a crucial role in marine ecosystems. Despite the extreme conditions of the deep-sea environment, they are capable of decomposing organic matter and transforming complex organic compounds into simpler ones, thereby providing energy for deep-sea ecosystems [4]. Additionally, microbes participate in the cycling of essential elements, which is vital for maintaining the stability and balance of deep-sea ecosystems [5,6]. Meanwhile, a large number of microorganisms may only exist in specific regions due to their specialized habitat requirements [7]. Therefore, a thorough understanding of the diversity and composition of deep-sea sediment microbial communities and their assembly mechanisms is a prerequisite for elucidating deep-sea ecosystem functions.

Community assembly refers to the processes that regulate the formation and maintenance of communities, and a thorough understanding of these mechanisms is essential for elucidating microbial ecological functions [8,9,10]. Community assembly is typically driven by a combination of deterministic processes (e.g., environmental filtering and biotic interactions) and stochastic processes (e.g., random birth–death and ecological drift) [11,12]. Niche theory and neutral theory are two major complementary frameworks for explaining the process of community assembly [8]. Niche theory emphasizes deterministic processes, proposing that environmental filtering and species-specific niche differences lead to community differentiation. In contrast, neutral theory assumes ecological equivalence among species at the same trophic level and attributes community assembly primarily to stochastic processes, such as ecological drift and dispersal, rather than niche-based selection [13,14,15]. Within the Western Pacific Ocean, several studies have focused on the microbial diversity in deep-sea sediments (e.g., the Yap Trench, Mariana Trench, and the Western Pacific Warm Pool) [16,17,18,19,20]. However, few studies have investigated the microbial community assembly processes and their driving factors in such habitats.

The influence of environmental factors on microbial communities is particularly critical for understanding community assembly mechanisms in deep-sea sediments. Generally, geochemical and biotic processes are tightly coupled in aquatic environments and can regulate species distribution and abundance, thereby influencing overall ecosystem functioning [21,22,23]. High hydrostatic pressure (HHP) exerts significant inhibitory effects on microbial physiological activities by acting on molecular structures, primarily manifested in membrane fluidity, DNA replication, RNA synthesis, protein synthesis, motility, nutrient uptake, and cell division [24,25,26]. Additionally, heavy metals pose a significant threat to the functions of microbial communities. Studies indicate that metals could bind to biomolecules on the surface of microbial cells, interfering with enzyme activity and normal metabolic processes, thus inhibiting microbial growth and reproduction [27]. The toxicity of heavy metals can also lead to an increase in populations of metal-resistant microbes, reducing community diversity [28]. Therefore, sedimentary environments with HHP and metal-rich conditions may affect the process of community assembly and drive the formation of unique microbial communities.

The Philippine Sea is an important area for studying marine environmental changes due to its complex bathymetry, hydrology, geological history, and diverse biogeochemical processes. This region is adjacent to the Indo-Pacific Convergence Zone (IPCZ), which plays a key role in mass transport between ocean basins [29]. In this region, although numerous studies have focused on the effects of heavy metals on macrofauna, research specifically investigating their impact on sedimentary microbial communities remains relatively limited [30,31]. As marine engineering such as mining and oil and gas extraction progressively advances into deeper waters, understanding the effects of different environmental factors on deep-sea microbes is crucial for protecting the health of deep-sea ecosystems.

This study explored bacterial diversity and composition, community assembly, and their relationship with environmental factors in deep-sea sediments of the Philippine Sea. First, we investigated both the horizontal and vertical distribution of bacterial communities by high-throughput 16S rRNA gene sequencing. Then, we applied the neutral community model [32] and the null model [11] to reveal the bacterial community assembly mechanisms. Finally, we evaluated the differential influence of environmental factors on the formation of bacterial community diversity in deep-sea sediments.

## 2. Materials and Methods

### 2.1. Sample Collection

Sample collection was conducted aboard R/V Dayang in January 2024 during Cruise DY84. One sediment core was collected from each of the four stations (MC01, MC02, MC03, and MC07) using a gravity corer. Three cores (MC01, MC02, and MC03) were collected from the central Parece Vela Basin from 6 to 9 January 2024, whereas MC07 was collected from the West Philippine Basin on 25 January 2024. Sampling locations and detailed station information are shown in Figure 1 and Table S1.

Each sediment core was sectioned at 2 cm intervals, except for the terminal sections of MC01 and MC02, which were 3 cm thick. MC01, MC02, MC03, and MC07 comprised 27, 25, 21, and 26 depth intervals, respectively. A single sediment sample was analyzed for each depth interval. Subsamples intended for microbial community analysis were stored at −80 °C until DNA extraction, whereas the remaining material was freeze-dried for subsequent physicochemical analyses, including heavy metals, total organic carbon (TOC), total organic nitrogen (TON), grain size, and water content (WC).

### 2.2. Measurement of Sediment Physicochemical Parameters

The determination of heavy metal content in sediment samples was conducted following the method described by Wang et al. [33]. First, the sediment samples were freeze-dried and homogenized by grinding. Subsequently, 0.2 g of the pretreated sample was accurately weighed and subjected to acid digestion using a mixture of 6 mL nitric acid, 2 mL hydrochloric acid, and 1 mL hydrofluoric acid until no white residue was visible. The digested sample was then transferred to a microwave digestion system (Multiwave 3000, Anton Paar, Graz, Austria) for further processing. Following microwave digestion, the sample was placed in an acid dryer and slowly evaporated to a volume of approximately 0.5 mL. The sample was then diluted to 10 mL with ultrapure water and filtered through a 0.22 μm membrane filter. Finally, heavy metal content was analyzed using an inductively coupled plasma mass spectrometer (Agilent 7900, Agilent, Santa Clara, CA, USA).

The determination of TOC and TON in sediment samples was conducted as follows: first, 1 g of homogenized sediment sample was placed in a centrifuge tube and treated with 30 mL of 2.4 mol/L hydrochloric acid for 16 h to remove inorganic carbon. Subsequently, the sample was separated by centrifugation and washed with ultrapure water. This washing process was repeated five to six times to thoroughly remove residual acid. The washed samples were then dried to a constant weight in an oven at 60 °C. The dried samples were carefully wrapped in tinfoil and analyzed for organic matter content using an elemental analyzer (Flash 2000 CHNS/O, Thermo Fisher Scientific, Waltham, MA, USA). Each sample was measured in triplicate to ensure reliability, with the absolute error of measurements maintained within ±0.3%. The final results are expressed as percentages of dry weight (%). The method for determining sediment WC and grain size is described in Text S1.

### 2.3. DNA Extraction, PCR Amplification, and 16S rRNA Gene Sequencing

The genomic DNA was extracted from the sediment samples using the TGuide S96 Magnetic Soil DNA Kit (Tiangen Biotech Co., Ltd., Beijing, China) according to the manufacturer’s instructions. Then, the DNA concentration of the sediment samples was measured with the Qubit dsDNA HS Assay Kit and Qubit 4.0 Fluorometer (Invitrogen, Thermo Fisher Scientific, Eugene, OR, USA). To determine the bacterial community diversity and structure, the 27F (AGRGTTTGATYNTGGCTCAG) and 1492R (TASGGHTACCTTGTTASGACTT) universal primer set was used to amplify the full-length 16S rRNA gene from the genomic DNA extracted from each sample. After successful amplification, SMRTbell libraries were prepared from the amplified DNA using the SMRTbell Express Template Prep Kit 2.0 according to the manufacturer’s instructions (Pacific Biosciences, Menlo Park, CA, USA). Purified SMRTbell libraries from the pooled and barcoded samples were sequenced on a single PacBio Sequel II 8M Cell using the Sequel II sequencing kit 2.0.

### 2.4. Sequence Analysis

The sequence analysis was performed using the BMK Cloud (Biomarker Technologies Co., Ltd., Beijing, China). The raw reads generated from sequencing were filtered and demultiplexed using the SMRT Link software (version 8.0) with minPasses ≥ 5 and minPredictedAccuracy ≥ 0.9, in order to obtain the circular consensus sequencing (CCS) reads. Subsequently, lima (version 1.7.0) was employed to assign the CCS sequences to the corresponding samples based on their barcodes. CCS reads lacking primers and those outside the length range (1200–1650 bp) were discarded through primer recognition and quality filtering using the Cutadapt quality-control procedure (version 2.7). The UCHIME algorithm (v8.1) was used to detect and remove chimera sequences to obtain the clean reads. Sequences with similarity ≥ 97% were clustered into the same operational taxonomic unit (OTU) by USEARCH [34] (v10.0), and the OTUs that appeared only once (singletons) were filtered. Taxonomy annotation of the OTUs was performed based on the Naive Bayes classifier in QIIME2 [35] using the SILVA database (release 138) [36] with a confidence threshold of 70%.

### 2.5. Bioinformatics and Statistical Analyses

Prior to diversity analyses, the OTU table was rarefied to the minimum sequencing depth of 31,915 reads per sample. Alpha diversity indices, including OTU richness, ACE, Chao1, Simpson, Shannon–Wiener, Faith’s phylogenetic diversity (Faith’s PD), and Good’s coverage, were calculated using QIIME2 (version 2020.6). Bray–Curtis dissimilarities were calculated to evaluate beta diversity, and principal coordinate analysis (PCoA) was performed based on the Bray–Curtis dissimilarity matrix. Differentially abundant bacterial taxa among stations were identified using linear discriminant analysis effect size (LEfSe), implemented in the microeco package (version 1.13.0) in R (version 4.4.2). Taxa with an LDA score > 4 were considered biomarkers.

Microbial co-occurrence networks were constructed separately for each sediment core using the 300 most abundant OTUs as candidate taxa. Pairwise associations among OTUs were evaluated using Spearman’s correlation, and correlations with |r| ≥ 0.6 and Benjamini–Hochberg-adjusted *p* < 0.01 were retained for network construction. OTUs without any retained associations (degree = 0) were removed from the final networks. Pairwise correlation analysis and network construction were performed using the Hmisc (version 5.2-2) and igraph (version 2.1.4) packages in R (version 4.4.2). Network visualization and the calculation of node-level topological features, including degree, closeness centrality, and betweenness centrality, were performed using Gephi 0.10.1.

Community assembly processes were evaluated using both the neutral community model (NCM) proposed by Sloan et al. [32] and a phylogenetic null-model framework. The NCM was used to characterize the relationship between OTU occurrence frequency and relative abundance. Model fit was evaluated using the coefficient of determination (R^2^), and the parameter Nm, representing the product of community size (N) and migration rate (m), was used as an indicator of dispersal capacity.

Phylogenetic community assembly was further evaluated using the beta nearest taxon index (βNTI) and the Raup–Crick metric based on Bray–Curtis dissimilarity (RCBray). Values of |βNTI| > 2 indicated a predominant role of deterministic processes, with βNTI < −2 indicating homogeneous selection and βNTI > 2 indicating heterogeneous selection. For pairwise comparisons with |βNTI| < 2, RCBray < −0.95 indicated homogeneous dispersal, RCBray > 0.95 indicated dispersal limitation, and |RCBray| < 0.95 indicated undominated processes.

Relationships between bacterial communities and environmental variables were evaluated using canonical correspondence analysis (CCA). Environmental variables with variance inflation factor (VIF) values > 10 were excluded prior to ordination analysis. Detrended correspondence analysis (DCA) indicated a longest gradient > 4; therefore, CCA was selected. The relationship between phylogenetic turnover (βNTI) and Fe dissimilarity was further explored using Pearson’s correlation and linear regression. Correlations among heavy metals were assessed using Pearson’s correlation. Additional computational details are provided in Text S2.

## 3. Results

### 3.1. Physicochemical Characteristics of Sediments

Heavy metal concentrations showed marked variation among the four sediment cores (Figure S1 and Table S2). Overall, MC03 and MC07 exhibited higher metal concentrations than MC01 and MC02. Metal nodules were observed in the surface (0–2 cm) sediments of MC03. Fe was the dominant metal across the sediment samples, accounting for approximately 89.2% of the total measured metal content, followed by Mn at 9.9%.

### 3.2. Distribution Patterns of Alpha and Beta Diversity in Sediment Bacterial Communities

After quality filtering and rarefying the raw data to the minimum sample size of 31,915 sequences, a total of 5,965,065 high-quality reads were obtained from 99 sediment samples. At a 97% similarity threshold, these sequences were clustered into 29,030 OTUs, which were taxonomically assigned to 41 phyla, 100 classes, 370 orders, 577 families, 1345 genera, and 3213 species. The rarefaction and Shannon diversity curves of all samples tended to plateau (Figure S2). Good’s coverage ranged from 0.93 to 0.99, with an average of 0.98, indicating that the sequencing depth was sufficient to capture the bacterial diversity present in the samples.

Stations MC02 and MC03, located at relatively shallow depths (4932 m and 4876 m, approximately 5000 m), exhibited higher alpha diversity values than the deeper stations MC01 and MC07 (6346 m and 5936 m, approximately 6000 m) (Table 1). However, OTU richness and Chao1 at MC02 were slightly lower than those at MC07. In sediments from all stations, bacterial alpha diversity values generally showed a decreasing trend with increasing sediment depth, with most indices reaching their maximum values within the top 10 cm (Figure S3).

Principal coordinate analysis (PCoA) based on Bray–Curtis dissimilarities showed distinct clustering patterns among samples from the four sediment cores (Figure 2a). Samples from MC02 and MC03 clustered relatively closely, whereas some samples from MC01 and MC07 showed partial overlap. Within the MC01 core, samples from shallow sediments (0–16 cm) and deep sediments (22–55 cm) formed two distinct clusters in the PCoA. Separately, within-core Bray–Curtis dissimilarities were higher in MC02 and MC07 than in MC01 and MC03 (Figure 2b).

### 3.3. Bacterial Community Composition and Vertical Distribution Patterns

A total of 41 bacterial phyla were identified in the deep-sea sediments of the Philippine Sea. Proteobacteria dominated the bacterial communities across all stations, representing the highest relative abundance at 35.4% of all sequences. The average relative abundance of Proteobacteria exceeded 20% at all stations, reaching up to 43% at MC02. The ten most abundant phyla were Proteobacteria (35.4%), Firmicutes (10.8%), unclassified Bacteria (9.7%), Chloroflexi (7.9%), Actinobacteriota (4.6%), Planctomycetota (4.3%), Verrucomicrobiota (4.0%), Acidobacteriota (3.8%), Bacteroidota (3.8%), and Gemmatimonadota (2.1%), which together accounted for 86.7% of all sequences. Within Proteobacteria, Gammaproteobacteria (49.8%) and Alphaproteobacteria (50.2%) were represented in almost equal proportions.

Bacterial community composition and relative abundance exhibited distinct patterns with sediment depth. Proteobacteria were the most common phylum across all layers at the four stations. However, in some layers of MC01 and MC07, unclassified Bacteria and Firmicutes showed higher relative abundances (Figure 2c). At MC01, unclassified Bacteria reached 20.1% and displayed a sharp increase in relative abundance at 18 cm depth, surpassing Proteobacteria to become the dominant group. Aerophobota were mainly detected below 18 cm depth but were nearly absent in surface sediments. The bacterial community composition of each layer at MC02 and MC03 stations was relatively stable, but there was some variation in relative abundance. At MC07, Firmicutes outcompeted Proteobacteria at several depths (0–6 cm, 18–30 cm, and 46–58 cm).

Kiloniellaceae and Magnetospiraceae exhibited distinct vertical variation patterns across the sediment cores (Figure S4). At MC01 and MC02, the vertical distributions of these two families showed patterns corresponding to variations in Fe concentrations (Figure 1 and Figure S4).

Exploratory LEfSe analysis revealed taxa showing differential relative abundance among samples from the four sediment cores (Figure 3). Chloroflexi, Acidobacteriota, Aminicenantales, and Aerophobota were more abundant in MC01 samples; Actinobacteriota and Proteobacteria in MC02; Nitrospirota, Myxococcota, and Planctomycetota in MC03; and Bacteroidota in MC07.

### 3.4. Species Co-Occurrence Patterns and Community Assembly Processes in Sediments

Co-occurrence network analysis revealed marked differences in bacterial community interactions among sites (Figure 4a). The networks at MC01 and MC03 contained more nodes and denser connections, with MC01 exhibiting more negative associations. In contrast, the MC02 network contained considerably fewer connected nodes and edges. Comparisons of node-level topological properties further showed that nodes at MC01 and MC03 had higher degree and betweenness centrality values but lower closeness centrality values than those at MC02 (Figure 4b). At a finer taxonomic level, OTUs affiliated with Kiloniellaceae and Magnetospiraceae were identified among the key nodes in these networks (Figure 4a). At the phylum level, the taxonomic composition of keystone nodes varied among sites: Proteobacteria, Chloroflexi, and unclassified Bacteria at MC01; Proteobacteria at MC02; Proteobacteria and unclassified Bacteria at MC03; and Proteobacteria and Firmicutes at MC07. These results suggested substantial differences in network complexity, interaction patterns, and keystone taxa among bacterial communities from different sites.

The neutral community model (NCM) successfully estimated the relationship between OTU occurrence frequency and relative abundance (Figure 5a). The model explained 65.4% and 58.5% of the variation at the shallower sites MC02 and MC03 (~5000 m), respectively, but only 22.2% and 26.4% at the deeper sites MC01 and MC07 (~6000 m). In the model, the Nm value, defined as the product of species abundance (N) and community migration rate (m), represented the dispersal capacity of species among communities. The Nm values of bacterial communities at sites MC02 and MC03 (673 and 1523, respectively) were higher than those at the deeper sites MC01 and MC07 (418 and 396, respectively).

The βNTI results indicated that bacterial communities in all four sediment cores were predominantly governed by stochastic processes, with deterministic processes accounting for only 12.3–21.0% (Figure 5b). In MC01, no homogeneous selection was detected, whereas heterogeneous selection accounted for 20.2%, the highest proportion among the four sediment cores. Homogeneous dispersal (32.4%) and dispersal limitation (35.0%) contributed nearly equally. In MC02, homogeneous dispersal accounted for 57.7%, whereas dispersal limitation accounted for only 4.7%. In MC03 and MC07, the relative contributions of the different ecological processes were more evenly distributed (Figure 5b).

### 3.5. Environmental Factors Affecting Bacterial Community Composition and Assembly

Significant associations were detected between bacterial community composition and several measured environmental variables. Canonical correspondence analysis (CCA) showed that bacterial community composition in MC01 and MC07 was associated with water depth and WC, whereas samples from MC02 and MC03 were mainly associated with Fe, Cu, Mn, Pb, Cr, TOC, TON, and Md. The explanatory rate of the CCA was 28.89% (Figure 6a). An exploratory regression analysis showed a positive relationship between Fe dissimilarity and βNTI (R^2^ = 0.25; Figure 6b).

## 4. Discussion

### 4.1. Bacterial Richness and Diversity in Deep-Sea Sediments of the Philippine Sea

Compared with studies from other deep-sea regions, the present results revealed higher bacterial diversity, which may be attributed to the use of long-read sequencing technology. Third-generation sequencing on the PacBio Sequel II platform enables full-length sequencing of bacterial 16S rRNA genes, allowing for more comprehensive taxonomic identification and more accurate estimates of alpha and beta diversity, thereby improving the resolution of distribution patterns of sediment bacterial communities at fine spatial scales [37]. The large proportion of unclassified Bacteria detected at site MC01 further suggested the presence of many unknown bacterial taxa awaiting exploration. Overall, the community composition at the phylum level across sites was consistent with previous findings from deep-sea sediments, being dominated by Proteobacteria, Firmicutes, Chloroflexi, Actinobacteriota, and Planctomycetota [16,38,39]. Along the vertical profiles of sediment cores, bacterial community composition showed variations likely driven by microenvironmental differences. The alpha diversity indices of all four cores decreased with depth, with many indices peaking near the surface. This trend was consistent with earlier reports and may be related to redox gradients within the sediments [18,40]. Our study highlighted the importance of micro-scale, high-resolution sampling in deep-sea sediments to more accurately resolve community diversity and vertical distribution patterns.

### 4.2. Bacterial Community Features Potentially Associated with Chemosynthetic Environments in the Parece Vela Rift

MC01 is located within the fracture zone of the Parece Vela Rift, where the geological structure is highly complex (Figure S5). This core exhibited pronounced vertical differences between shallow (0–16 cm) and deep (22–55 cm) sediments, as reflected by beta-diversity patterns and bacterial community composition. Previous studies have reported that Aerophobota were significantly enriched in hydrate-bearing sediments of the South China Sea, particularly in silt-dominated layers, but were nearly absent in sandy sediments [41,42,43]. Additionally, Dehalococcoidia were capable of anaerobic methane oxidation [44], while Aminicenantales, also enriched at this site, have been detected in marine sediments, crustal fluids, and cold seeps [45,46]. These microbial groups to some extent reflected the environmental characteristics of site MC01. Although the maximum water depth of the Parece Vela Rift approaches 7000 m, far deeper than the continental margin environments where chemosynthetic communities were typically found (e.g., South China Sea, 1120–1350 m) [47,48], recent discoveries of cold seep communities in the Kuril–Kamchatka Trench and the western Aleutian Trench at depths up to 9553 m suggested that chemosynthesis-based ecosystems may be far more widespread than previously anticipated [49]. Taken together, these observations suggest that MC01 may harbor environmental conditions compatible with chemosynthetic microbial processes, although direct geochemical and geological evidence is currently lacking.

### 4.3. Community Assembly Mechanisms and Environmental Associations in Deep-Sea Sediments

CCA showed that bacterial community composition varied along several measured environmental gradients. In addition, the exploratory regression indicated an apparent association between Fe dissimilarity and phylogenetic turnover. Among the six measured metals, Fe and Mn accounted for 89.2% and 9.9% of the total, respectively. This phenomenon can be attributed to the location of the study area within the Pacific “Ring of Fire”, where volcanic activity along coastal and submarine volcanoes produces large amounts of lava and volcanic ash. These Fe- and Mn-rich materials accumulated on the seafloor through sedimentary processes, contributing substantially to marine iron and manganese cycling [50,51]. All metals measured in this study are classified as heavy metals. Under long-term exposure to heavy metal-rich sedimentary environments, some bacteria gradually evolved resistance under selective pressure, exhibiting adaptive capacity to heavy metals. In contrast, other bacteria were more sensitive to heavy metal stress, limiting their survival and reproduction [52,53]. Taken together, these patterns suggest that heavy metal concentrations and other geochemical parameters may be associated with variation in sediment bacterial communities. Fe showed a particularly strong association with phylogenetic turnover.

The vertical distributions of Kiloniellaceae and Magnetospiraceae also showed correspondence with Fe concentrations, particularly in MC01 and MC02. Kiloniellaceae are chemoheterotrophic bacteria with denitrification potential and have been detected in marine basalts and metalliferous environments, suggesting a possible association with metal cycling [54,55,56]. Magnetospiraceae include magnetotactic bacteria with biomineralization and Fe-scavenging capabilities and are commonly associated with Fe–Mn-rich sediments [57]. These patterns suggest that both groups may be involved in Fe cycling within the sampled sediments, although their specific ecological roles require further investigation.

The relatively sparse co-occurrence network observed in MC02 may reflect weaker co-variation among dominant taxa across sediment depth intervals, consistent with the comparatively stable vertical community composition in this core. Because the network was constructed from the 300 most abundant OTUs and only associations meeting the predefined thresholds (r ≥ 0.6, *p* < 0.01) were retained, the lower number of connected nodes in MC02 should not be interpreted as lower bacterial richness. Rather, it indicates that fewer dominant OTUs exhibited strong and significant co-occurrence patterns across the sampled depth intervals.

The composition and biogeographic patterns of microbial communities are shaped by both stochastic and deterministic processes, and quantifying their relative contributions to community assembly remains a key challenge in microbial ecology [10,12,58]. This study analyzed the assembly mechanisms of sediment bacterial communities using the NCM and βNTI, but the two approaches yielded somewhat different results. NCM showed lower model fits in the two deeper cores (MC01 and MC07) than in the two shallower cores (MC02 and MC03), whereas βNTI indicated that stochastic processes predominated across all four sediment cores. These differences arise from the distinct assumptions and focal points of the two approaches. The NCM assumes that community assembly is shaped by dispersal and ecological drift, with a primary focus on species abundance within communities [32]. In contrast, βNTI excludes the effects of stochastic ecological processes such as dispersal and ecological drift, assumes that species are not functionally equivalent, and considers environmental selection as the primary driver of community assembly [58]. Although horizontal gene transfer (HGT), which is common among microorganisms, may not necessarily disrupt ecological niches among species [11], in many extreme environments strongly constrained by nutrient metabolism (e.g., deep sea, saline–alkali soils, and the oceanic crust), environmental selection pressures may fail to leave sufficient and reliable phylogenetic signals in the 16S rRNA gene. Taken together, these results indicate differences in community assembly patterns among the four sampled sediment cores. At the phylogenetic level, stochastic processes predominated across all cores, whereas the apparent differences related to water depth require validation using spatially replicated sampling.

A limitation of the present study is that only one sediment core was collected from each station; therefore, the depth intervals within each core do not constitute independent field replicates at the station level. Consequently, differences observed among the four sediment cores should be interpreted as patterns specific to the sampled cores rather than as replicated estimates of spatial variation among stations. Future studies incorporating replicate sediment cores at each station will be necessary to rigorously evaluate spatial variability in bacterial communities and their assembly processes.

## 5. Conclusions

This study analyzed the composition and distribution of bacterial communities in deep-sea sediments of the Philippine Sea, as well as the co-occurrence patterns of important taxa. It also revealed the community assembly mechanisms in deep-sea metal-rich sediment environments and the factors influencing them. The three main conclusions are as follows:

(1) Bacterial diversity showed distinct patterns among the four sampled sediment cores. The two shallower cores generally exhibited higher alpha diversity values than the two deeper cores, although this pattern was not consistent for all indices. Within individual cores, bacterial diversity generally declined with increasing sediment depth.
(2) The distinct bacterial community observed in the deep sediments of MC01, together with the occurrence of taxa previously reported from hydrate- and seep-associated environments, suggests that the Parece Vela Rift may contain conditions potentially favorable for chemosynthetic microbial processes. This hypothesis requires verification through direct geological, geochemical, and in situ observations.
(3) Stochastic processes predominated in community assembly across the four sampled sediment cores. Differences in NCM parameters and βNTI-derived assembly processes were observed among cores, while sediment Fe showed an apparent association with phylogenetic turnover. Because one sediment core was collected at each station, differences among cores should be regarded as descriptive patterns requiring validation with spatially replicated sampling.

## Figures and Tables

**Figure 1:**
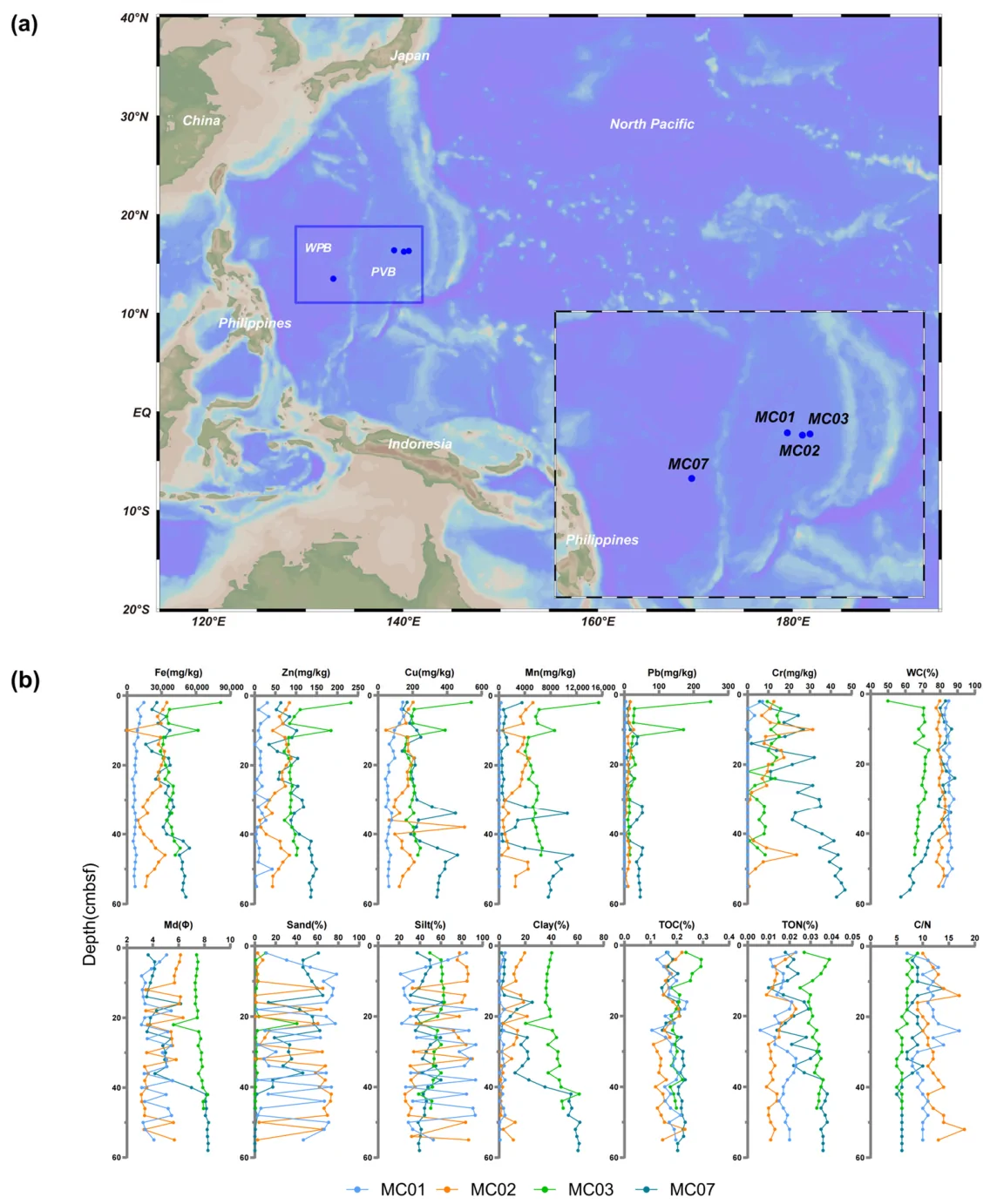
Geographic locations of the sampling stations and vertical profiles of sediment environmental parameters in the Philippine Sea: (**a**) Locations of the four sampling stations (MC01, MC02, MC03, and MC07). (**b**) Vertical profiles of Fe, Zn, Cu, Mn, Pb, Cr, water content (WC), median grain size (Md), sand, silt, clay, total organic carbon (TOC), total organic nitrogen (TON), and carbon-to-nitrogen ratio (C/N). Sediment depth is expressed as centimeters below the seafloor (cmbsf), and Md is expressed on the phi (Φ) scale. Colors indicate different sampling stations.

**Figure 2:**
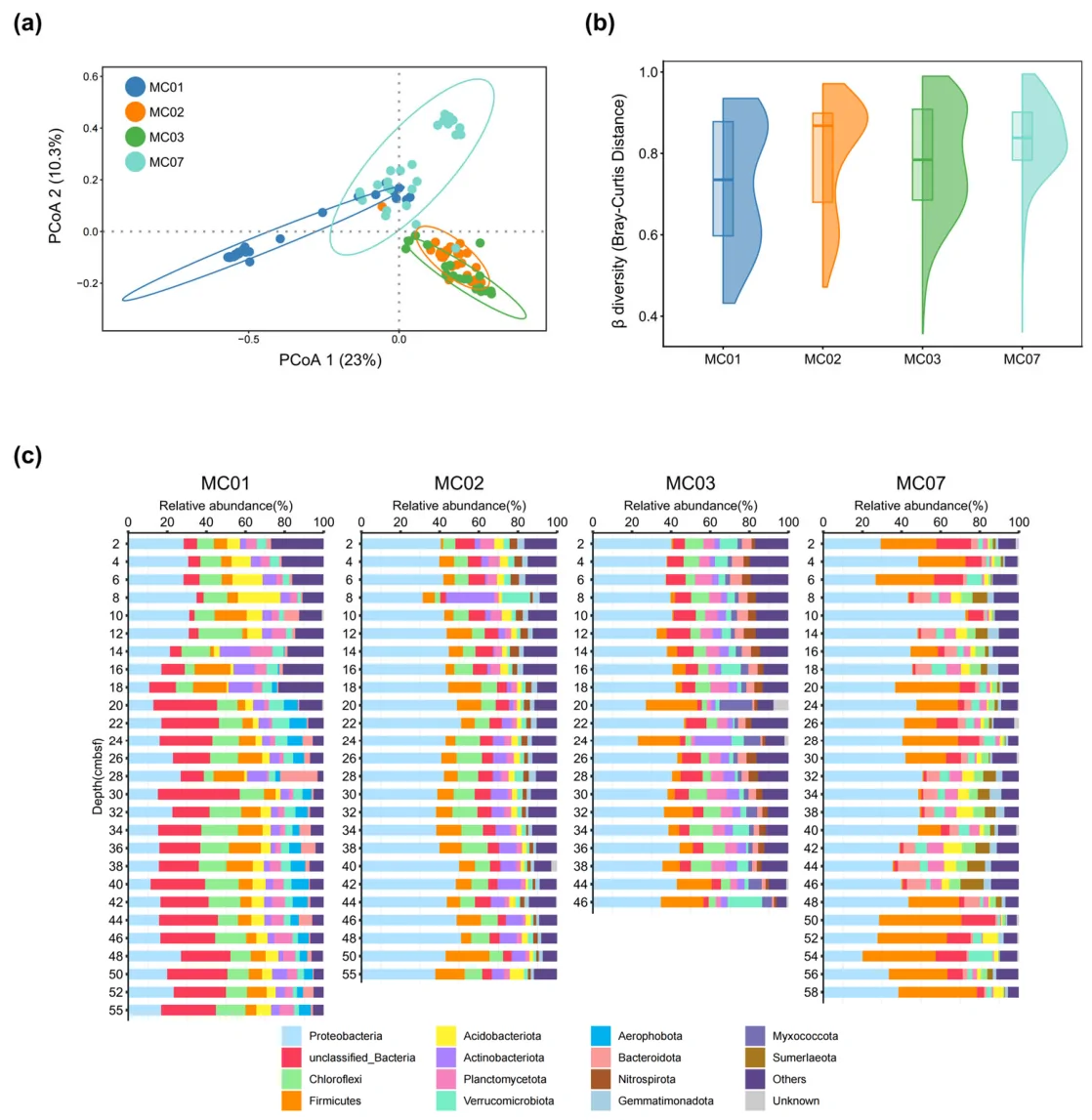
The diversity, composition, and abundance of bacterial communities in sediments from the Philippine Sea: (**a**) Principal coordinate analysis based on the Bray–Curtis dissimilarity. (**b**) Distribution of within-core Bray–Curtis dissimilarities for the four sediment cores. (**c**) Bacterial community compositions at the phylum level and their relative abundance.

**Figure 3:**
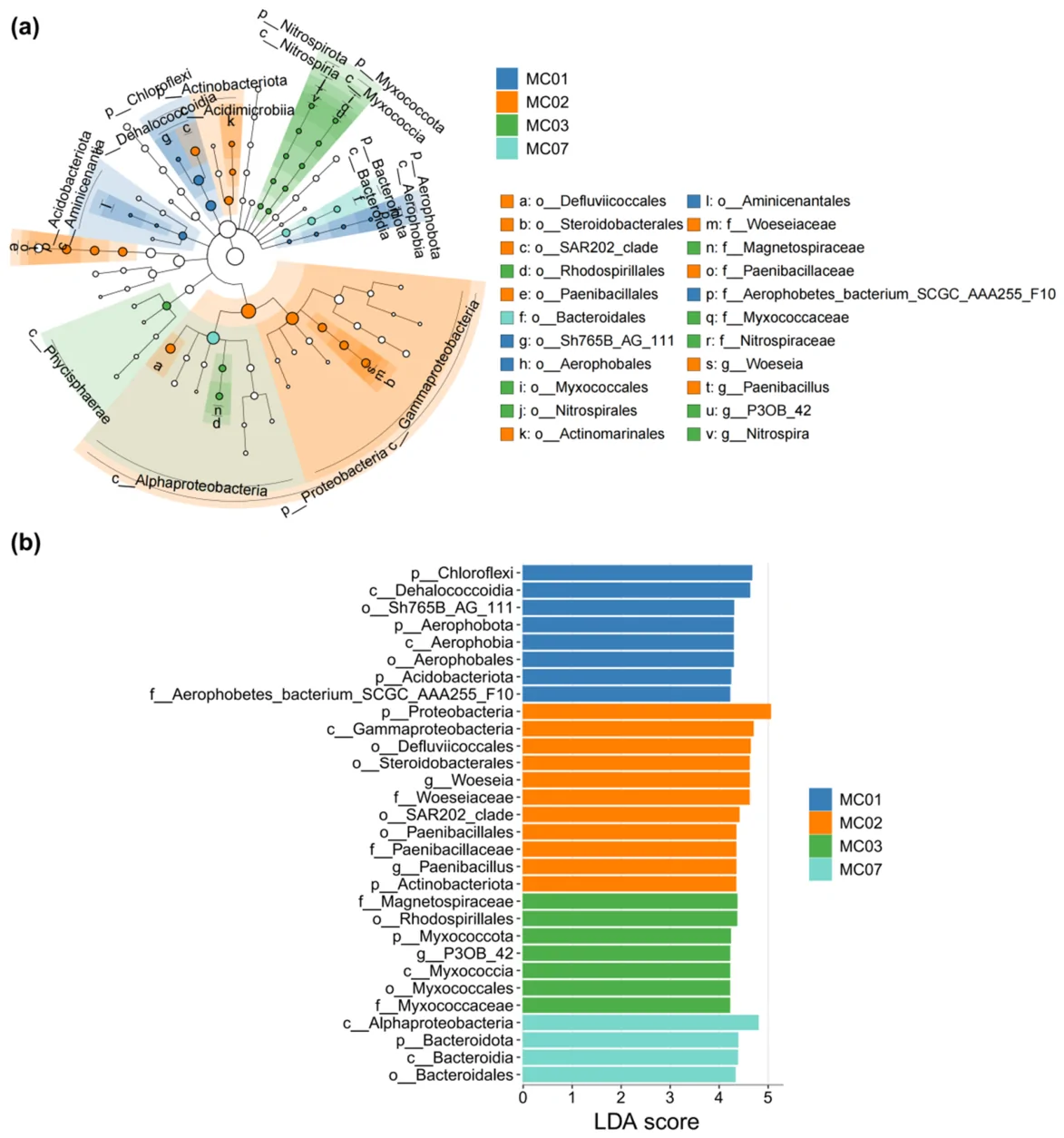
Exploratory LEfSe analysis of bacterial taxa showing differential relative abundance among samples from the four sediment cores: (**a**) Cladogram indicating the phylogenetic distribution of bacterial communities (200 taxa, 50 features). (**b**) Bar plot showing the distribution of the linear discriminant analysis (LDA) values (TOP30 and only bacterial families with LDA score > 4 are shown).

**Figure 4:**
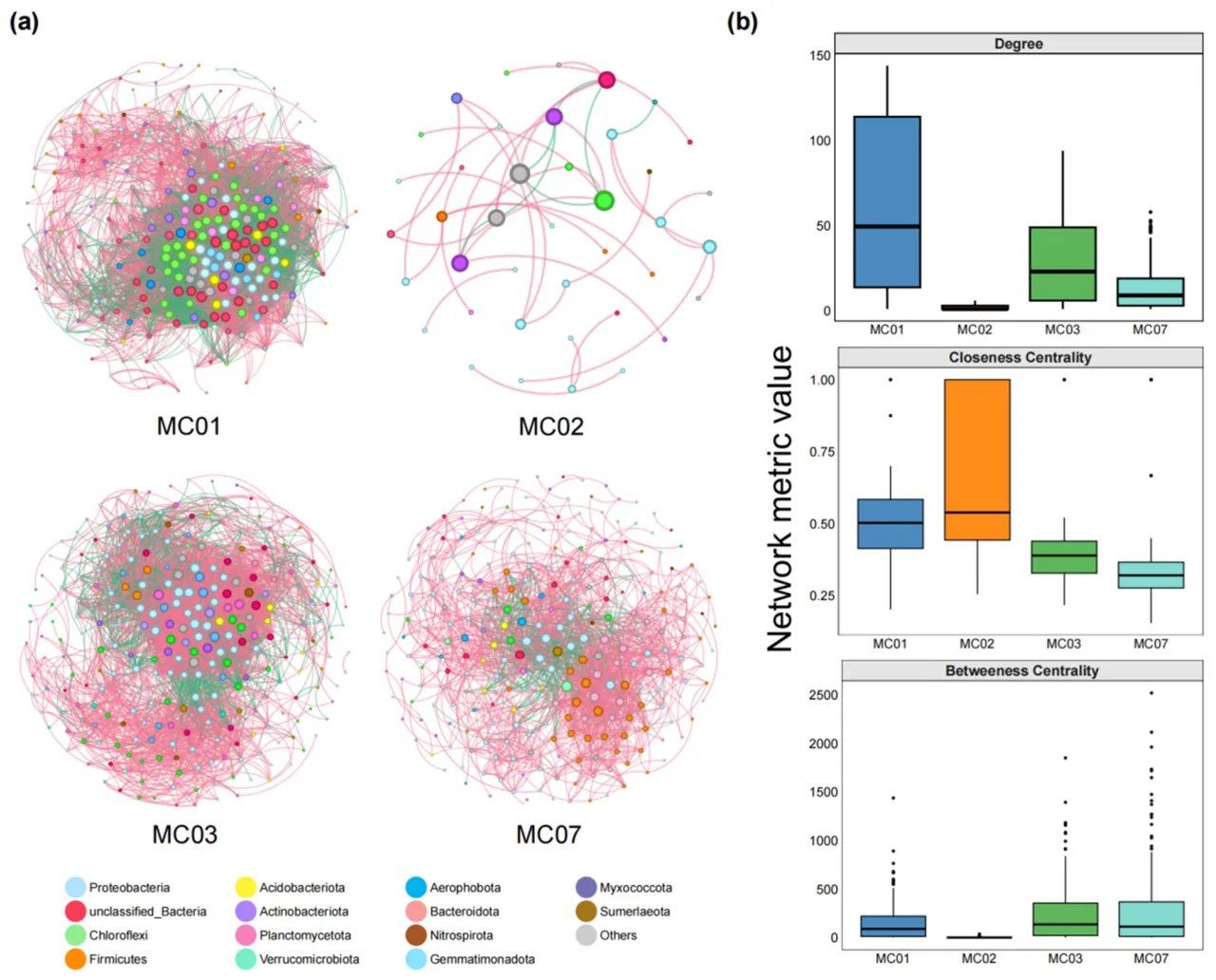
Co-occurrence networks of the bacterial community in all stations and their characteristics: (**a**) Microbial co-occurrence networks with OTUs colored by taxonomy (phylum level) for each station. Each displayed node has a Spearman correlation coefficient greater than 0.6 and a *p*-value less than 0.01. The size of the node represents the number of connections. (**b**) Node-level network topological features of the bacterial community from each station.

**Figure 5:**
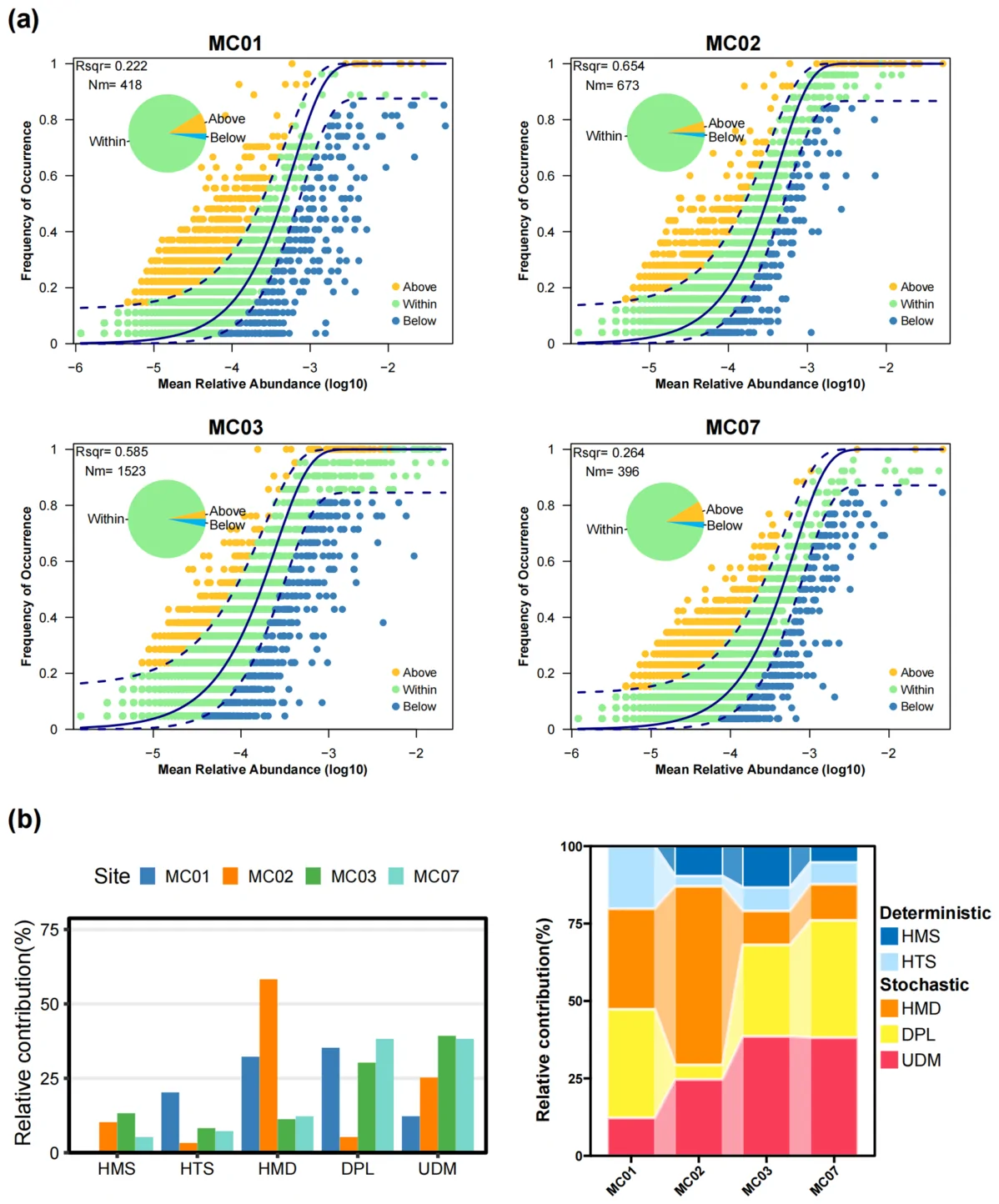
The community and spatial distribution patterns of bacterial communities in sediments from the Philippine Sea: (**a**) The predictions of the neutral community model (NCM). The solid blue line represents the best fit of the neutral community model, and the dashed blue lines indicate the 95% confidence intervals. Green dots denote taxa that fit the model predictions (mainly influenced by stochastic drift), orange dots represent taxa occurring at higher frequencies than predicted (typically widespread species), and blue dots represent taxa occurring at lower frequencies than predicted (typically rare species). (**b**) Community assembly mechanisms explained by the null model. The model categorized community assembly mechanisms into five ecological processes. Deterministic processes included homogeneous selection (HMS) and heterogeneous selection (HTS), while stochastic processes included homogeneous dispersal (HMD), dispersal limitation (DPL), and undominated processes (UDM).

**Figure 6:**
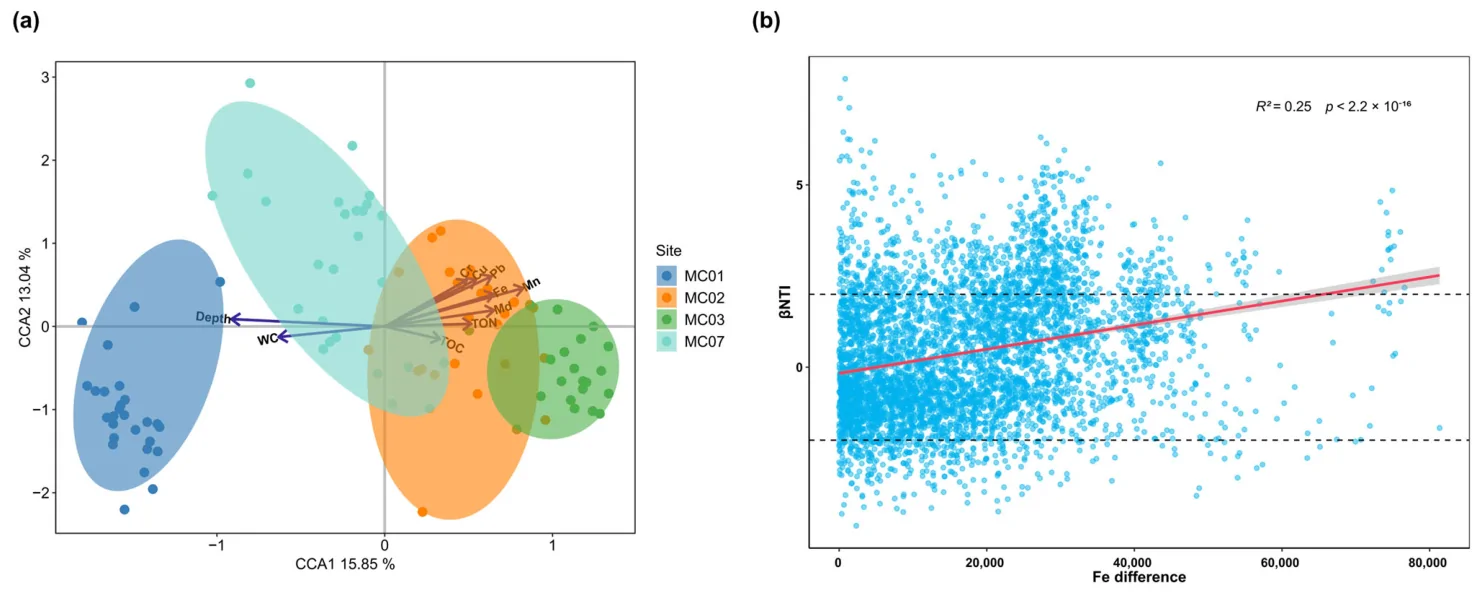
Relationships between bacterial communities and measured environmental variables: (**a**) Canonical correspondence analysis (CCA) showing associations between bacterial community composition and measured environmental variables. TOC, total organic carbon; TON, total organic nitrogen; WC, water content; Md, median grain size. (**b**) Exploratory relationship between βNTI and Fe dissimilarity. In (**b**), blue dots represent pairwise sample comparisons, dashed horizontal lines indicate the βNTI thresholds of ±2, the solid red line represents the fitted linear regression, and the gray shading denotes the 95% confidence interval.

**Table 1:** Average bacterial alpha diversity of 4 sediment cores from the Philippine Sea. PD: phylogenetic diversity of the whole tree.

Station	OTU	ACE	Chao1	Simpson	Shannon	PD	Coverage
MC01	1194	2051	1847	0.9758	7.18	22.80	0.9874
MC02	1306	2572	2215	0.9865	7.87	25.60	0.9860
MC03	2080	3231	3011	0.9898	8.63	28.19	0.9780
MC07	1440	2482	2227	0.9723	7.46	22.79	0.9839

## Data Availability

The data presented in this study are available from the first author or either of the corresponding authors upon reasonable request. The data are not publicly available due to project confidentiality requirements. The environmental data supporting the reported results are provided in the Supplementary Materials.

## Supplementary Materials

The following supporting information can be downloaded at https://www.mdpi.com/article/10.3390/microorganisms14102129/s1, Text S1. Analysis of water content and grain size in deep-sea sediments from the Philippine Sea; Text S2. The bioinformatics analysis of the bacterial community; Table S1. Information on sediment sample collection stations in the Philippine Sea; Table S2. Heavy metal concentrations and physicochemical properties of deep-sea sediments from the Philippine Sea; Figure S1. Metal concentrations and their correlations with physicochemical parameters in deep-sea sediments from the Philippine Sea; Figure S2. Rarefaction curves and Shannon curves of all sediment samples; Figure S3. Depth chart of alpha diversity indices for each station; Figure S4. Vertical distribution of Kiloniellaceae and Magnetospiraceae in the sediment cores. (a) Station MC01. (b) Station MC02. (c) Station MC03. (d) Station MC07; Figure S5. Location of station MC01 on the GEBCO 2025 Grid. References [59,60] is cited in the Supplementary Materials.
