# Supplementary material for: Bacterial Diversity and Community Assembly in Deep-Sea Sediments: Observations from the Philippine Sea
Source: Microorganisms. 2026 Sep 22;14(10):2129. doi: 10.3390/microorganisms14102129 (PMC13707928; doi:10.3390/microorganisms14102129)
Supplement: Supplementary file 1 [file microorganisms-14-02129-s001.zip › microorganisms-4533625-supplementary.pdf]

Supplementary Information

Bacterial Diversity and Community Assembly in Deep-Sea Sediments: Observations from the Philippine Sea

Cheng Yang[1,2], Dong Sun[3], Yongji Huang[4], Mingyan Lai[1,2], Li Zhang[5,6], Hao Yin[1], Yanqun Li[1], Lina Lyu[1,*], Si Zhang[1,4,*]

[1] State Key Laboratory of Tropical Oceanography, South China Sea Institute of Oceanology, Chinese Academy of Sciences, Guangzhou 510301, China

[2] University of Chinese Academy of Sciences, Beijing 100049, China

[3] Key Laboratory of Marine Ecosystem Dynamics, Second Institute of Oceanography, Ministry of Natural Resources, Hangzhou 310012, China

[4] Southern Marine Science and Engineering Guangdong Laboratory (Guangzhou), Guangzhou 511458, Guangdong, China

[5] Guangxi Key Laboratory of Beibu Gulf Marine Resources, Environment and Sustainable Development, Fourth Institute of Oceanography, Ministry of Natural Resources, Beihai 536000, China

[6] Key Laboratory of Tropical Marine Ecosystem and Bioresource, Fourth Institute of Oceanography, Ministry of Natural Resources, Beihai 536000, China

*Corresponding author:

Lina Lyu and Si Zhang

South China Sea Institute of Oceanology, Chinese Academy of Sciences

164 Xingang Road, Guangzhou 510301, China.

E-mail address: lvlina@scsio.ac.cn; zhsimd@scsio.ac.cn.

Content in Supporting Information

Page

Texts

Text S1. Analysis of water content and grain size in deep-sea sediments from the Philippine Sea. S3

Text S2. The bioinformatics analysis of bacterial community. S4

Tables

Table S1. Information on sediment samples collection stations of the Philippine Sea. S7

Table S2. Heavy metal concentrations and physicochemical properties of deep-sea sediments from the Philippine Sea. S8

Figures

Figure S1. Metal concentrations and their correlations with physicochemical parameters in deep-sea sediments from the Philippine Sea. S9

Figure S2. Rarefaction Curves and Shannon Curves of all sediment samples S10

Figure S3. Depth chart of Alpha diversity indices for each station S11

Figure S4. Vertical distribution of Kiloniellaceae and Magnetospiraceae in the sediment cores. (a) station MC01. (b) station MC02. (c) station MC03. (d) station MC07. S12

Figure S5. Location of station MC01 on the GEBCO 2025 Grid S13

Text S1. Analysis of water content and grain size in deep-sea sediments from the Philippine Sea.

The method for determining sediment water content was as follows: First, weighed the wet sediment sample and recorded its initial weight. Then, placed the sample in an oven at 105°C and dried it to a constant weight, recording the dry weight. The water content of the sediment was calculated using the following formula:

$WC=(M_w-M_d)/M_w \times 100\%$

M_W : wet weight; M_d : dry weight; WC: water content.

The sediment grain size analysis was conducted as follows: First, the sediment samples were thoroughly mixed and dried at 80°C. 1 g of the dried sample was placed in a beaker and soaked in distilled water. 0.05 mol/L sodium hexametaphosphate was added into beaker as a dispersing agent. After standing for 24 hours, the sample was sonicated for 15 minutes to ensure complete particle dispersion. Subsequently, the sample was analyzed using a laser particle size analyzer (Mastersizer2000G, Malvern Panalytical, UK) with a measurement range of 0.02-200 μm. Each sample was measured in triplicate, with the relative error maintained within 2%. The analysis results included parameters of median grain size (Md), mean grain size (Mz), sand content, silt content, and clay content.

Text S2. The bioinformatics analysis of bacterial community.

Alpha-diversity indices were assessed for each sample using QIIME2 (version 2020.6). The calculated indices included OTU richness, ACE (abundance-based coverage estimator), Chao1, Simpson's index, Shannon–Wiener index, PD whole tree (Faith's phylogenetic diversity) and Coverage. Bray–Curtis distances were calculated using the vegan package (version 2.6-10) in R (version 4.4.2) to evaluate the beta diversity of bacterial communities, and principal coordinate analysis (PCoA) was performed based on the Bray–Curtis dissimilarity matrix. Biomarker microorganisms in sediments were identified using linear discriminant analysis (LDA) effect size (LEfSe) implemented in the microeco package (version 1.13.0) in R (version 4.4.2).

Microbial co-occurrence network analysis was performed based on community data obtained from high-throughput sequencing. Co-occurrence network analysis was conducted in R using the Hmisc (version 5.2-2) and igraph (version 2.1.4) packages to reflect microbial co-existence relationships in sediments. To simplify the analysis, the 300 most abundant OTUs were selected as candidate taxa for network construction. Pairwise correlations were calculated using Spearman's correlation, and associations with $|r| \geqslant 0.6$ and Benjamini－Hochberg-adjusted $p < 0.01$ were retained. OTUs with no retained associations (degree = 0) were removed from the final networks before subsequent topological analyses. The co-occurrence network was visualized in Gephi 0.10.1, which was also used to compute topological features of the nodes, including degree (the number of connections of a node), closeness centrality (the reciprocal of the average shortest path length from a node to all other nodes), and betweenness centrality (the frequency with which a node lies on the shortest paths between other nodes).

The neutral community model (NCM) for prokaryotic communities proposed by Sloan et al. [32] is commonly used to explain how stochastic and deterministic processes shape bacterial community assembly. Community data were analyzed in R using the Hmisc (version 5.2-2), minpack.lm (version 1.2-4), and stats4 (version 4.4.2) packages to construct the neutral community model. A higher R^2 value indicates closer agreement between the observed community data and the predictions of the neutral community model. The parameter N describes community size and represents species

abundance; m denotes migration rate, with higher values indicating greater ease of species dispersal; and Nm, the product of N and m, reflects the overall dispersal capacity of species among communities.

Phylogenetic community assembly was evaluated using the beta nearest taxon index (βNTI) and the Raup–Crick metric based on Bray–Curtis dissimilarity (RCBray). Pairwise beta mean nearest taxon distance (βMNTD) was calculated using the comdistnt function in the picante package (version 1.8.2) in R (version 4.4.2). The βNTI values were obtained by standardizing the observed βMNTD values against null distributions generated from 999 randomizations. Values of $|\beta NTI| > 2$ indicated deterministic processes, with $\beta NTI < -2$ indicating homogeneous selection and $\beta NTI > 2$ indicating heterogeneous selection. Values of $|\beta NTI| < 2$ indicated stochastic processes, which were further partitioned using RCBray. The RCBray metric was calculated by comparing the observed Bray–Curtis dissimilarities with their null distributions. For pairwise comparisons with $|\beta NTI| < 2$, RCBray < -0.95 indicated homogeneous dispersal, RCBray > 0.95 indicated dispersal limitation, and $|RCBray| < 0.95$ indicated undominated processes, such as ecological drift.

Canonical correspondence analysis (CCA) was applied to assess relationships between measured environmental variables and bacterial communities. Variance inflation factor (VIF) analysis was performed, and variables with VIF values > 10 were excluded. Detrended correspondence analysis (DCA) indicated a gradient length > 4; therefore, CCA was performed using the vegan package (version 2.6-10) in R (version 4.4.2).

To explore the relationship between phylogenetic turnover and Fe variation, pairwise differences in Fe concentration were regressed against βNTI values, and the relationship was evaluated using Pearson's correlation and linear regression.

Table S1. Information on sediment samples collection stations of the Philippine Sea.

Station	Latitude (°N)	Longitude (°E)	Depth (m)	Core length (cm)	Sampling method
MC01	16.4	139.1	6346	55	gravity corer
MC02	16.2	140.1	4932	55	gravity corer
MC03	16.3	140.6	4876	46	gravity corer
MC07	13.5	132.9	5936	58	gravity corer

Table S2. Heavy metal concentrations and physicochemical properties of deep-sea sediments from the Philippine Sea. Median grain size (Md) was given in Φ; Water content (WC), sand, silt, clay, total organic carbon (TOC), and total organic nitrogen (TON) contents were expressed as percentages (%). Heavy metal concentrations were reported in mg/kg.

Station	Fe	Zn	Cu	Mn	Pb	Cr	WC	Md	Sand	Silt	Clay	TOC	TON
**MC01**													
Mean±SD	7671.1±2119.8	13.7±10.9	76.7±29.8	88.6±57.7	0.8±1.5	0.4±1.5	84.7±1.4	4.2±0.9	42.1±30.3	56.0±28.8	1.8±1.6	0.18±0.03	0.02±0.00
Range	4789.5-14681.0	0.0-42.1	43.3-149.0	46.9-343.7	0.0-4.5	0.0-7.2	81.7-87.9	3.1-5.5	1.9-78.3	21.3-94.0	0.3-4.6	0.10-0.24	0.01-0.02
**MC02**													
Mean±SD	23295.2±8465.3	58.0±21.5	163.2±79.4	2634.8±1560.3	10.5±5.9	7.8±7.7	80.5±1.7	4.7±1.2	33.7±31.8	57.9±25.6	8.5±6.8	0.16±0.03	0.01±0.00
Range	44.7-34605.0	12.3-88.0	45.6-500.8	44.6-5256.3	0.6-26.9	0.7-31.3	77.7-84.3	3.1-6.3	0.3-73.2	25.7-90.0	1.1-21.4	0.11-0.23	0.01-0.02
**MC03**													
Mean±SD	39568.1±11258.7	99.2±36.3	219.3±83.4	6059.8±2259.5	35.4±56.7	8.8±4.9	68.2±4.6	7.5±0.5	3.7±8.4	55.5±6.7	41.0±7.6	0.21±0.03	0.03±0.00
Range	29160.3-81395.4	71.4-232.6	154.2-539.6	4068.7-15435	5.9-248.9	0.0-16.0	50.0-73.5	5.6-8.3	0.5-40.3	38.7-63.4	20.2-61.3	0.17-0.29	0.03-0.04
**MC07**													
Mean±SD	37055.7±10064.3	100.2±32.1	260.6±91.2	3468.1±3724.5	26.3±17.1	27.9±13	77.2±2.3	5.7±1.9	30.2±32.1	44.6±29.2	26.3±5.0	0.20±0.03	0.03±0.00
Range	16340.0-54293.2	34.6-148.9	140.2-460.2	338.8-11388.9	3.8-52.5	1.9-46.8	57.4-88.4	3.5-8.3	0.0-65.1	31.0-61.6	1.3-61.8	0.14-0.23	0.01-0.04

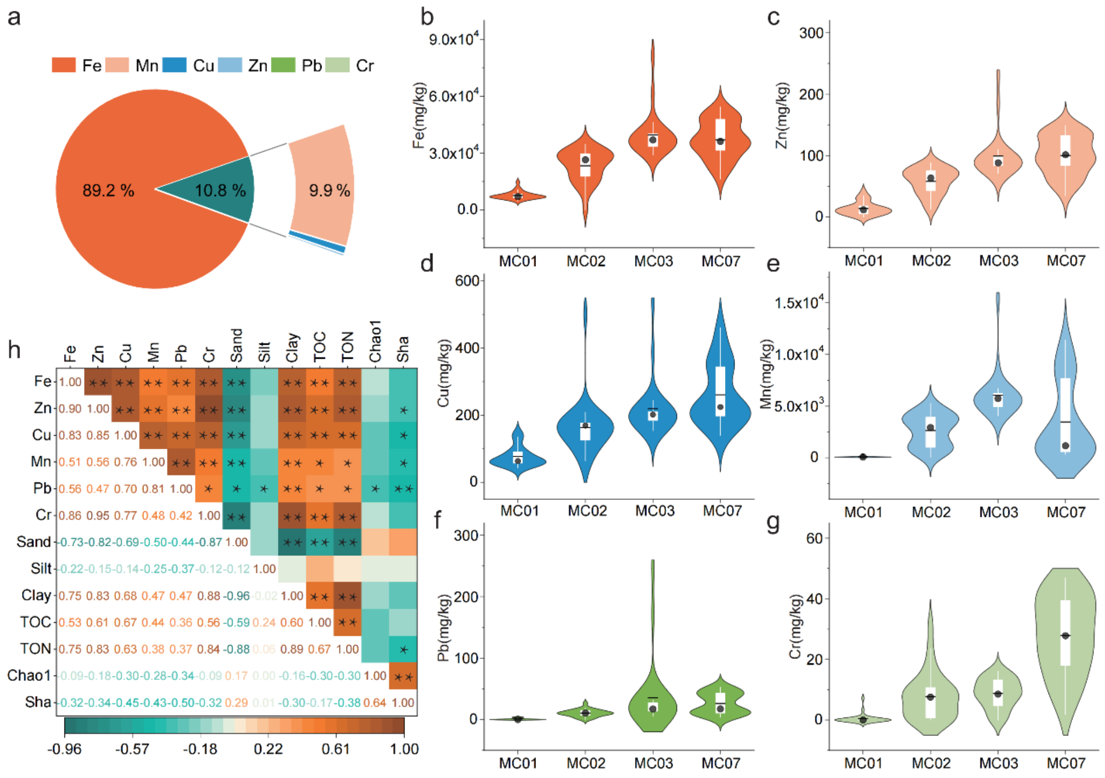

Figure S1. Metal concentrations and their correlations with physicochemical parameters in deep-sea sediments from the Philippine Sea. (a) Proportions of individual heavy metals relative to the total heavy metal content across all sediment samples. (b-g) Average concentrations of Fe, Zn, Cu, Mn, Pb, and Cr in the sediment cores, with black dots and short lines indicating the median and mean values, respectively. (h) Spearman correlation analysis between heavy metals and physicochemical parameters, with $^{**}p < 0.01$ and $^{*}p < 0.05$.

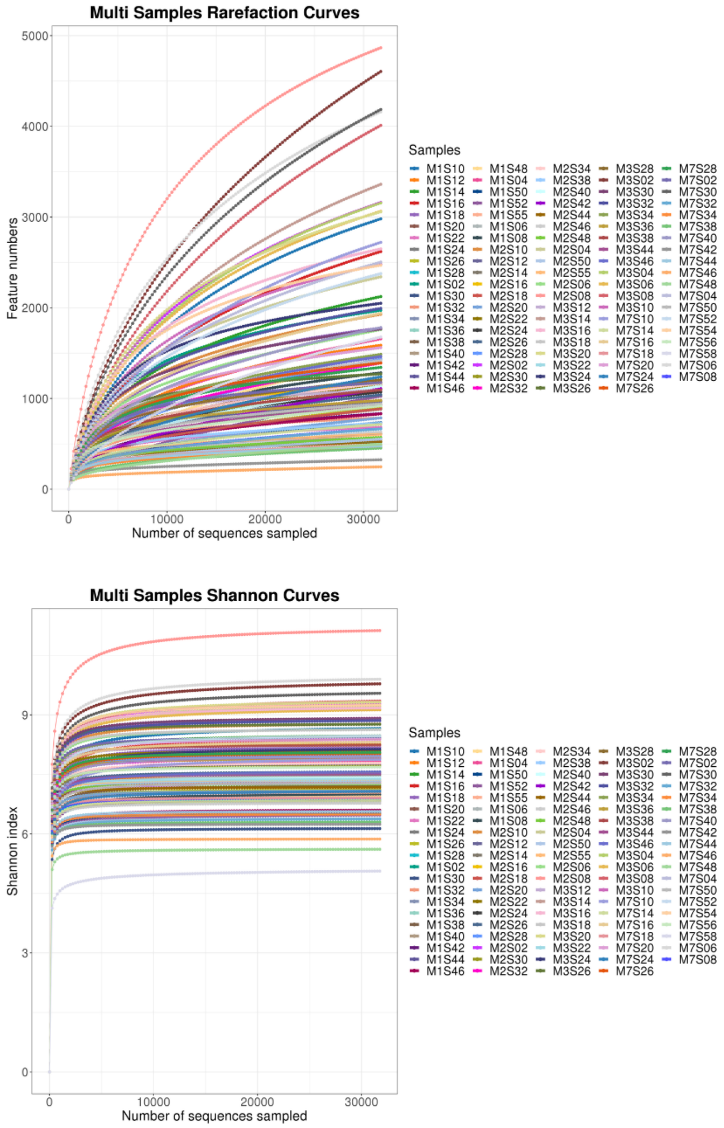

Figure S2. Rarefaction Curves and Shannon Curves of all sediment samples.

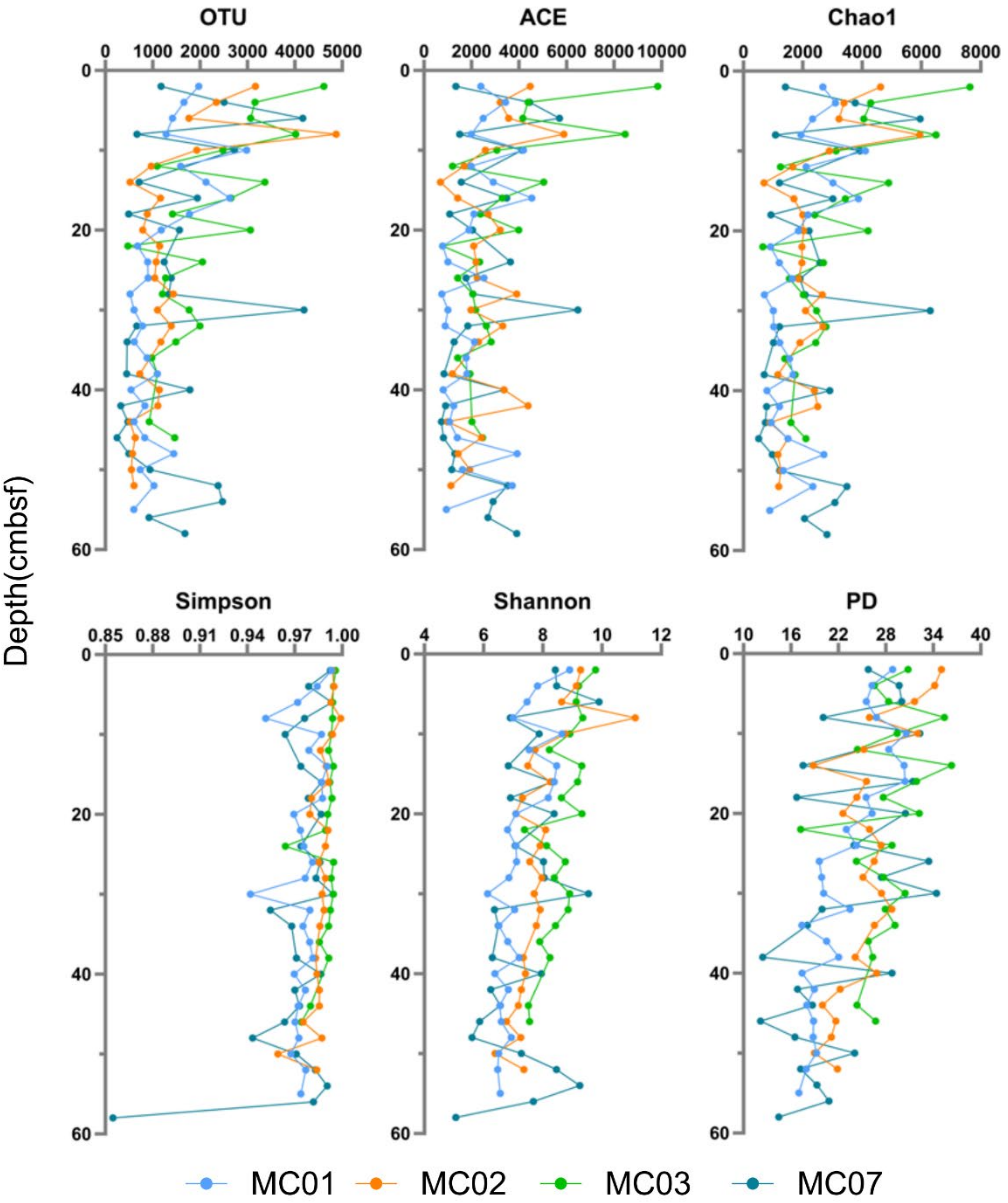

Figure S3. Depth chart of Alpha diversity indices for each station

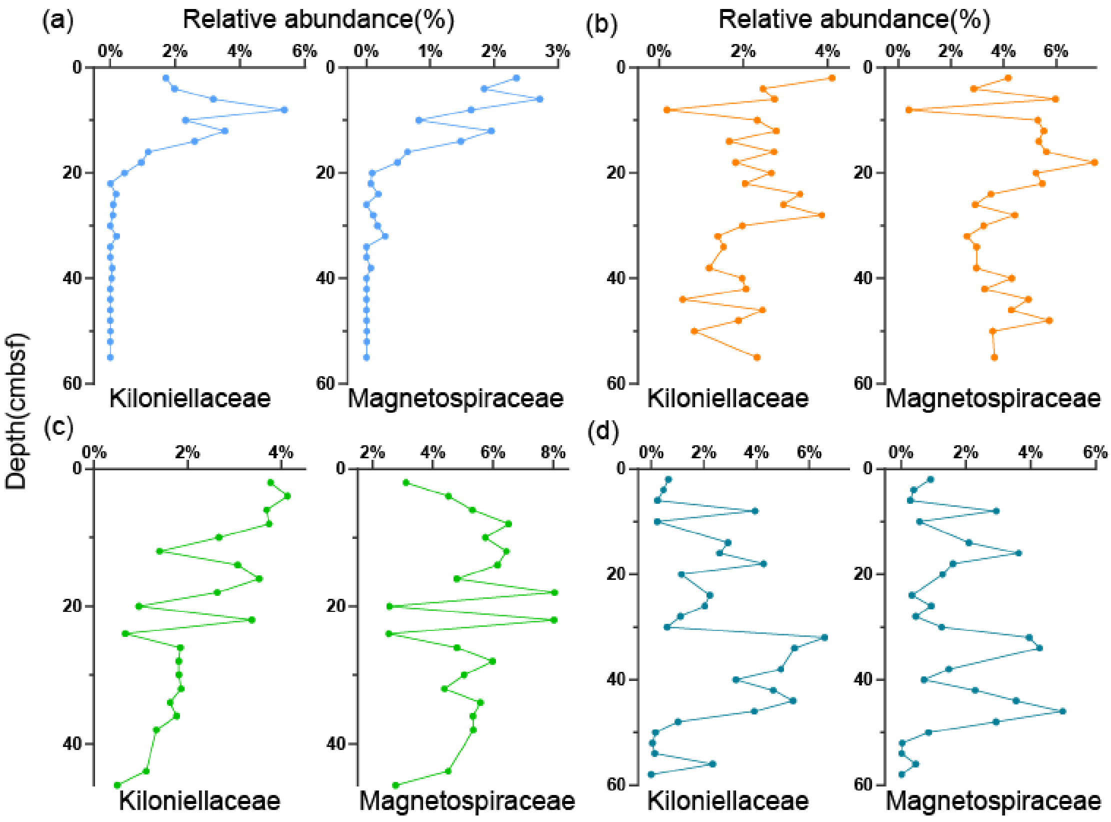

Figure S4. Vertical distribution of Kiloniellaceae and Magnetospiraceae in the sediment cores. (a) station MC01. (b) station MC02. (c) station MC03. (d) station MC07.

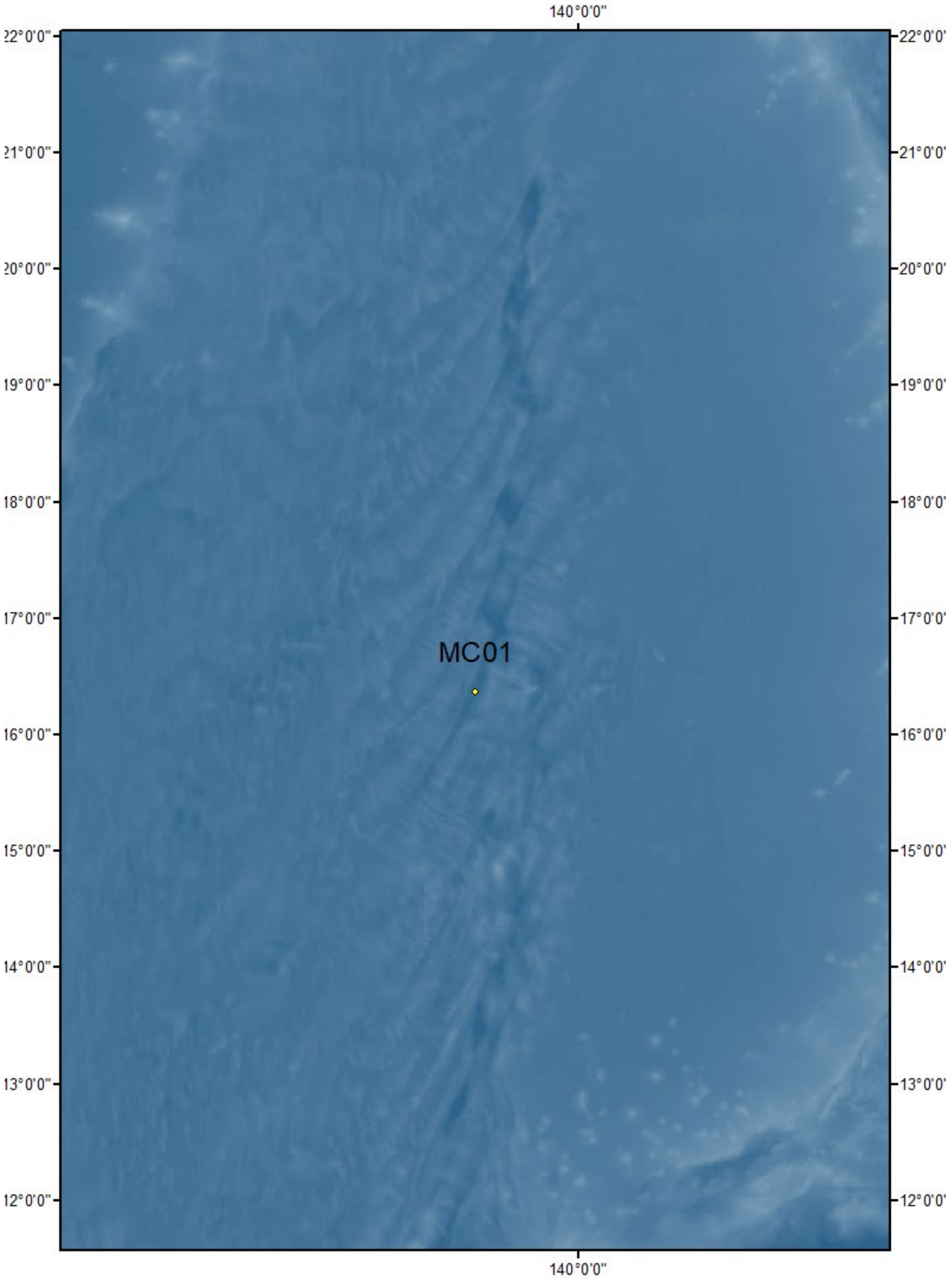

Figure S5. Location of station MC01 on the GEBCO 2025 Grid [59].
